# Patient-reported outcome measures in individuals with amelogenesis imperfecta: a systematic review

**DOI:** 10.1007/s40368-022-00737-3

**Published:** 2022-07-27

**Authors:** S. B. Appelstrand, A. Robertson, N. Sabel

**Affiliations:** 1 Public Dental Service, Region Västra Götaland, Västra Götaland, Sweden; 2 Department of Pediatric Dentistry, Institute of Odontology, Sahlgrenska Academy, University of Gothenburg, Göteborg, Sweden

**Keywords:** Amelogenesis imperfecta, Oral health-related quality of life, Patient-reported outcome measures, Esthetics, Self-esteem, Sensibility

## Abstract

**Purpose:**

Amelogenesis imperfecta (AI) is a hereditary condition which affects the composition and structure of enamel in terms of hypoplasia and/or hypomineralization. The condition severely affects patients facing such difficulties as hypersensibility, loss of tooth substance and poor aesthetics. The objective is to perform a systematic review of patient-reported outcome measures (PROMs) in patients with amelogenesis imperfecta.

**Methods:**

Inclusion criteria were articles written in English, including PROMs from patients with amelogenesis imperfecta. The databases PubMed, Scopus and Web of Science were searched on April 27, 2022, and eligible articles were screened. Exclusion criteria were articles based on proxy reports and single case reports.

**Results:**

405 studies were screened in terms of title and abstract, with 31 articles eligible for full-text screening, resulting in a total of 11 articles eligible for inclusion, (articles including 4–82 patients). The content was analyzed, resulting in the outcome divided into seven domains: Oral Health-Related Quality of Life (OHRQoL), Dental fear, Esthetics, Psychosocial factors, Function, Dental hypersensitivity, and Treatment outcome.

**Conclusion:**

The limited quantity of research on PROMS from patients with AI indicates a significant impact of OHRQoL and daily life. A large variety of approaches have been presented in the articles. Patients report concerns of esthetics, hypersensitivity, function, and a general impact on well-being and social interaction. This highlights the importance for the need of early dental treatment.

**Prospero registration number:**

256875.

## Introduction

Amelogenesis imperfecta (AI) is a hereditary condition which affects the composition and structure of enamel in terms of hypoplasia and/or hypomineralization. AI is present in both dentitions, even if not seen macroscopically, and varies in phenotype (Gadhia et al. 2012).

The clinical appearance ranges from smaller pits or grooves in the enamel surface to vast morphological disturbances with rough, discoloured and/or thin enamel (Sundell and Koch 1985).

The prevalence is between 1:700 and 1:14,000, depending on the study population (Crawford et al. 2007; Sundell and Koch 1985).

Researchers suggest several ways to classify AI, both in terms of inheritance pattern and clinical appearance. A well-recognized classification is that of Witkop and Rao from 1971, dividing AI into three main groups: *Hypoplastic*, *Hypocalcified*, and *Hypomature,* with several subcategories depending on genetics and phenotypes (Rao and Witkop 1971).

Another well-acknowledged classification system is that by Sundell and Koch involving *Hypoplastic* and *Hypomineralized*, each with subcategories based on phenotypes (Sundell and Koch 1985). Over the years the classification has been revised, become more descriptive and has come to include molecular defects (Aldred et al. 2003; Witkop 1988).

The hypoplastic form is a quantitative defect where enamel of reduced thickness and/or pitting and grooves are present.

The hypomineralized form is a qualitative defect, affecting the mineral components making the enamel weaker in structure and with a lower radiodensity, compared to sound enamel. Hypocalcified enamel appears opaque and is susceptible to discolouration and wear, whereas hypomature enamel is more speckled and softer, yet more resistant to attrition, compared to the hypocalcified type. Both hypoplastic and hypomineralized enamel can be seen in the same individual and even on the same tooth surface, making the clinical variation widespread (Gadhia et al. 2012; Sundell and Koch 1985).

The etiology of AI is genetic and caused by gene mutations, e.g., affecting the genes Amelogenin, Matrix metalloproteinaise-20, Enamelin, Kallikrein-4, or FAM83H (Gadhia et al. 2012; Wang et al. 2021; Xin et al. 2017). The formation of enamel is a complex process and alterations in the genetic encoding can lead to defects in the secretory stage, thin or hypoplastic enamel, or a defect maturation stage, with hypomature enamel as a result (Hu et al. 2007).

In addition to defective enamel, AI can include other dental co-morbidities such as malocclusion, attrition, delayed eruption, and deviant morphology (Poulsen et al. 2008).

Patients with AI often need extensive dental treatment from an early age. In the primary dentition, metal crowns may be placed on posterior teeth to prevent wear and decay. Additionally, anterior teeth may need restorations. As the permanent teeth erupt, long-term temporary treatment is performed gradually, facing difficulties such as hypersensibility, loss of tooth substance and concern about aesthetics, as well as coping strategies of the growing child (Crawford et al. 2007; McDonald et al. 2012). Treating the paediatric patient with AI may include several challenges for the dentist involving the individual handling of a young child and additionally, performing extensive, demanding treatments pain free at multiple visits (McDonald et al. 2012). The long-term treatment plan may involve fixed prosthodontics such as porcelain crowns (Crawford et al. 2007). Although temporary restorations are common today, recently published data highlight the possibilities of permanent crown therapy in early adolescence without severe complications (Lundgren and Dahllof 2014; Lundgren et al. 2018).

Patient-reported outcome measures (PROMs) are used for the assessment of quality and effectiveness in healthcare, reported by the patient. The use of PROMs assures a reduction of bias, since the reports come directly from the patient without interference from medical personnel or family members (Gilchrist and Marshman 2020).

Rare diseases and conditions affect the patient’s quality of life. Capturing the patient’s input and views ensures that the clinical decision-making is made with a patient-centered approach (Slade et al. 2018). In dentistry, as well as in many other fields, there are few standardised PROMs for measuring specific outcomes and there is a need for developing condition-specific instruments (Slade et al. 2018; Wittneben et al. 2018). In paediatric dentistry, there are several generic instruments for measuring oral health-related quality of life OHRQoL, such as Child Perception Questionnaire CPQ, Child Oral Health Impact Profile COHIP, and Early Childhood Oral Health Impact Scale ECOHIS (Gilchrist and Marshman 2020).

Since dental care should not only result in healthy oral conditions but also patient satisfaction including sustainability, aesthetics, and lack of symptoms, the use of PROMs is of great value.

Amelogenesis imperfecta is a rare condition greatly affecting the patient. In patients with AI, PROMs are of importance due to the characteristics of the disease and lifelong nature of the condition. The concerns for patients with AI have a range of issues including different aspects affecting daily life. Research highlights the importance of function, aesthetics, and psychosocial factors in young patients with AI, with the tooth shade being a strong variable (Parekh et al. 2014). Adolescents and adult patients with AI, using the Oral Health Impact Profile (OHIP) instrument, had experienced social disability, psychological discomfort and disability, and physical disability (Coffield et al. 2005; Hashem et al. 2013; Pousette Lundgren et al. 2015, 2016).

Today, approximately 1185 scientific articles are published concerning amelogenesis imperfecta. There are larger studies as well as case studies on clinical treatment, prosthodontic restorations, and choice of material to use. Systematic reviews are needed to accurately and reliably summarize evidence to outline the current research in a specific area.

The aim of this systematic review was to investigate self-reported oral health-related quality of life factors and patient-reported outcome measures (PROMs) in patients with amelogenesis imperfecta.

## Material and methods

**Fig. 1:**
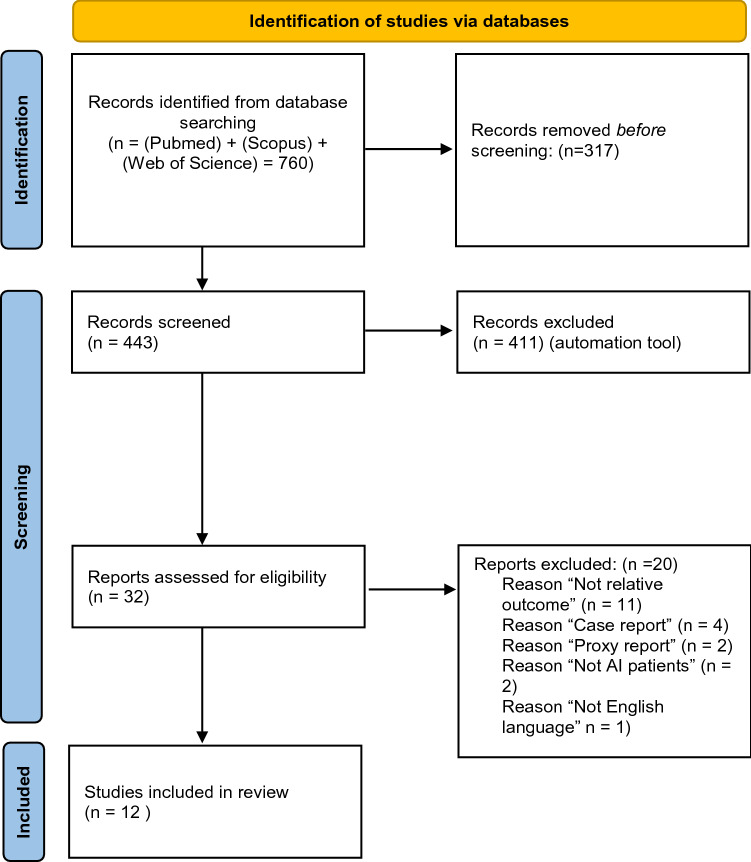
PRISMA flow diagram describing the flow of identification and selection of articles from the databases toward inclusion in the systematic review. From: Page et al. (2021)

The search was conducted as a systematic review of articles reporting on amelogenesis imperfecta and oral health-related quality of life parameters reported by the affected patients. The systematic steps were performed according to Moher et al. (2015). Literature search was performed in PubMed, Scopus, and Web of Science, with assistance from librarians HS and EH (Fig. 1).

PICO was performed to frame and answer the dental healthcare-related question of the project (Richardson et al. 1995).

- P—Patients with amelogenesis imperfecta.
- I—Investigate studies of patient reports in terms of oral health-related quality of life (OHRQoL), dental fear, aesthetics, psychosocial factors, function, dental hypersensitivity, and treatment outcome.
- C—Various control groups (before/after treatment, split mouth, healthy controls) or no controls.
- O—Summarized patient-reported outcome measures (PROMs).

### Search

**Fig. 2:**
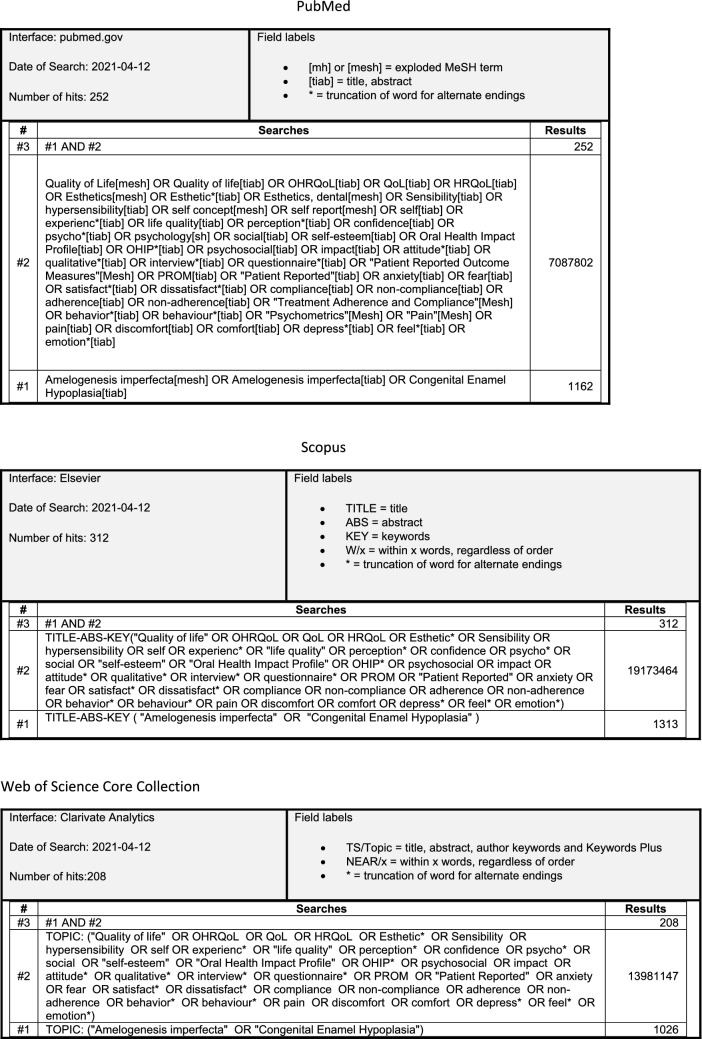
Search strategy in PubMed, Scopus and Web of Science Core Collection 2021-04-12, with the two combined blocks; Amelogenesis imperfecta and patient reports. The search strategy was re-ran April 2022. The updated search resulted in 38 new records and after screening 1 article was additionally included

The databases PubMed, Scopus, and Web of Science were searched for articles monitored by librarians (HS, EH) on April 27, 2021 with no limitation of publication year. Search block was performed as in Fig. 2, combining the two blocks amelogenesis imperfecta and PROMs.

### Selection

Articles were screened in terms of title and abstract by authors SB and NS separately and blinded, using the online software Rayyan Qatar Computing Research Institute (Ouzzani et al. 2016). Duplicates were eliminated before screening.

Studies that fulfilled the inclusion criteria, agreed by authors, were read in full text. The articles were reviewed separately by the authors. In case of disagreement, the articles were discussed between the authors, until agreement.

### Inclusion criteria

The inclusion criteria include full text scientific articles published in peer-reviewed journals. The subjects should be patients with amelogenesis imperfecta and the patient-reported outcome measures PROMs. The articles should be written in the English language.

### Exclusion criteria

Exclusion criteria include articles based on proxy reports, such as parental or dental personnel reports and articles based on single case reports.

Studies that fulfilled agreement between authors to be further included were read in full text. The articles were reviewed separately by the authors prior to inclusion. In case of disagreement, the articles were discussed between the authors (Fig. 1).

### Data extraction

Included articles were thoroughly reviewed and presented by country, study design, classification for AI diagnoses, number of participants, age, gender, control group, domains and instrument of PROMs, and results (Table 1).

**Table 1:** The study is represented by surname of the first author, year published and the abbreviation of the journal where the study is published is found in the table

Study	Year	Journal	Country	Study design	Ctrl	Domain	Instrument	*p* value
Chen	2013	Pediatr Dent	USA	Retro	B/A^†^	E^‡^	Questionnaire	*
						F^‡‡^	Questionnaire	ns
						DH^‡‡‡^	Questionnaire	*
Coffield	2005	JADA	USA	Cross	CC^††^	OHRQoL	OHIP-14	***
						E^‡^	Questionnaire	*
						PS^‡‡‡‡^	SAD	*
							FNE	*
							FAY	ns
							Rosenberg Self Esteem Scale	ns
Hashem	2013	J of Dent	Ireland		CC^††^	OHRQoL	OHIP-49	**
						PS^‡‡‡‡^	Rosenberg Self Esteem Scale	ns
Lindunger	2005	Int J Prosth	Sweden	Retro	–	E^‡^	Questionnaire	–
						TR^‡‡‡‡‡^	Questionnaire	–
Lyne	2021	Br Dent J	Great Britain	Cross	–	OHRQoL	Questionnaire	–
						E^‡^	Questionnaire	–
						DH^‡‡‡^	Questionnaire	–
						PS^‡‡‡‡^	Questionnaire	–
						TR^‡‡‡‡‡^	Questionnaire	–
Parekh	2014	Int J Paed Dent	Great Britain	Cross	–	OHRQoL	CPQ_11–14_	–
						E^‡^	Questionnaire	–
						PS^‡‡‡‡^	Questionnaire	–
						F^‡‡^	Questionnaire	–
						DH^‡‡‡^	Questionnaire	–
Pousette	2014	J of Dent	Sweden	Cross and retro	CC^††^	DH^‡‡‡^	VAS	***
Pousette	2015	Health and Qual Outcomes	Sweden	Cross	CC^††^	OHRQoL	OHIP-14	***
						DF^‡‡‡‡‡‡^	CFSS-DS	ns
						PS^‡‡‡‡^	DBS-R	ns
					B/A^†^	OHRQoL	OHIP-14	***
						DH^‡‡‡^	VAS	ns
Pousette	2016	PLOS ONE	Sweden		–	PS^‡‡‡‡^	Interviews	–
						DH^‡‡‡^	Interviews	–
						TR^‡‡‡‡‡^	Interviews	–
Pousette	2018	J of Dent	Sweden	Retro	B/A^†^	DH^‡‡‡^	VAS	***
Quandalle	2020	J of Appl Oral Scien	France	Cross	CC^††^	DH^‡‡‡^	Question	**
Sneller	2014	Int J Paed Dent	Great Britain	Cross	–	PS^‡‡‡‡^	Focus group	–

The study design is marked as “Cross” if cross-sectional designed and “Retro” if retrospective designed. Control group (Ctrl) is marked with B/A^†^ if the study persons were their own control group (before and after treatment), or CC^††^ if control group was included in the study. The domains investigated in the studies are seen as OHRQoL (Oral health-related quality of life), E^‡^ (Esthetics), F^‡‡^ (Function), DH^‡‡‡^ (Dental hypersensitivity), PS^‡‡‡‡^ (Psychosocial factors), TR^‡‡‡‡‡^ (Treatment results) and DF^‡‡‡‡‡‡^ (Dental fear). The instrument or methods for patient to report are listed as questionnaire (own constructed questionnaire), established questionnaires as OHIP-14, CPQ11–14, CFSS-DS, DBS-R are given by name, reports via VAS, Rosenberg Self Esteem Scale, SAD FAY, FNE, and via interviews or focus group. The significance, if reported, is presented *p > 0.05, **p > 0.01, ***p > 0.001 and below, as “ns” when no significance or “–” if no statistical calculations were presented

## Results

760 articles were found, resulting in 443 articles for review after deduplication. The 443 studies were screened in terms of title and abstract and 32 articles were eligible for full text screening. The articles were read by authors SB and NS separately and resulted in a total of 12 articles eligible for inclusion. Exclusion of articles was based on the inclusion and exclusion criteria (Fig. 1).

The 12 included articles displayed a great variability in terms of instruments for measuring patient-reported factors, as well as differences concerning population size and study characteristics. Ten of the articles were from Europe, including five from Sweden. The remaining two were from North America.

The studies were published during the past 16 years, with all but two in the last decade.

The studies showed a variety of designs and were performed with either a qualitative or quantitative approach (Table 1). Five studies were case–control studies with either a cross sectional or retrospective approach (Coffield et al. 2005; Hashem et al. 2013; Lundgren and Dahllof 2014; Pousette Lundgren et al. 2015; Quandalle et al. 2020). The case–control studies showed a great variety of instruments.

There were two classification systems noted for amelogenesis imperfecta (Rao and Witkop 1971; Sundell and Koch 1985).

Six articles used the Sundell and Koch classification (Lindunger and Smedberg 2005; Lundgren and Dahllof 2014; Lundgren et al. 2018; Pousette Lundgren et al. 2015, 2016) and three articles presented the Witkop classification (Chen et al. 2013; Quandalle et al. 2020; Sneller et al. 2014). Three studies did not clarify the system of classification. One article had genetically verified AI with no further specification (Coffield et al. 2005). The two remaining studies did not specify their way of classification (Hashem et al. 2013; Parekh et al. 2014). The hypoplastic form was found in 140 patients and the hypomature form of AI in 117 patients. The hypocalcified form of AI was seen in 42 patients, though not all studies specified classification (Table 2).

**Table 2:** Overview of patients in included studies

Study	Ages Years (mean)	*N*	M:F	Classification	Subtypes			
					HP	HC	HM	HP-HM
Chen	9–15 (13)	8	2:6	Witkop	4	2		2
Coffield	16–82 (37)	30	17:13	Genes		17		
Hashem	18–45	27	8:19	Not told				
Lindunger	14–37 (24)	15	7:8	Sundell	10		5	
Lyne	5–17 (12)^†^	60	20:38^†^	Sundell	21	9	16	18
Parekh	10–16 (32)	40	21:19	Not told				
Pousette 2014	6–25	82	40:42	Sundell	38		44	
Pousette 2015	6–25 (14)	69	33:36	Sundell	33		36	
Pousette 2016	16–23	7	2:5	Sundell	2		3	2
Pousette 2018	11–22	27	12:15	Sundell	15		12	
Quandelle	5–17 (11)	42	22:20	Witkop	14	14		14
Sneller	11–16 (14)	4	3:1	Witkop	3		1	

First author of the article is identifying the study

Ages of patients included in the studies (range and mean). Number of included patients are N, the number of males and females is found under column M:F

The system used for classification of amelogeneis imperfecta is declared for each study. The subtypes of AI are abbreviated as *HP* hypoplastic subtype, *HC* hypocalcified subtype, *HM* hypomature subtype, *HP–HM* combination of hypoplastic and hypomature subtype. ^†^Data missing from two of the included patients (age and sex)

### Study characteristics

A total of 411 (222 females, 187 males, 2 data missing) AI patients participated in the 12 selected studies. The number of participants in each article ranged from 4 to 82 with a median value of 28 participants. The ages spanned from 5 to 82 years, with most of the subjects being children or young adults. More than 80% of the participants were 25 years or younger. Three studies with a total of 72 patients had a wider age range: 16–82 years old, 18–45 years old, and 14–37 years old, respectively (Coffield et al. 2005; Hashem et al. 2013; Lindunger and Smedberg 2005) (Table 2).

### Qualitative and quantitative design

Three articles were of qualitative design: Two involved interviews (Parekh et al. 2014; Pousette Lundgren et al. 2016) and one involved focus group discussions (Sneller et al. 2014), all followed by thematic analysis.

The other articles had quantitative outcome data from the different instruments used.

One article used the Wong Baker FACES pain rating scale (Chen et al. 2013). One retrospective article used a questionnaire with VAS for rating the questions (Lindunger and Smedberg 2005). One article used no specific instrument, but asked patients *Do you feel pain when you eat, drink or brush your teeth?* with *Yes* or *No* answers (Quandalle et al. 2020).

One of the studies was a randomized controlled trial (RCT) measuring hypersensitivity with VAS before and after treatment (Lundgren et al. 2018).

The included articles investigated different aspects of PROMs. This review article categorises the aspects as different domains of PROMs. A list of domains and the instruments used for analyses is found in Table 2. Five articles had control groups (Coffield et al. 2005; Hashem et al. 2013; Lundgren and Dahllof 2014; Pousette Lundgren et al. 2015; Quandalle et al. 2020) and three articles investigated the studied group before and after treatment (Chen et al. 2013; Lundgren et al. 2018; Pousette Lundgren et al. 2015). All articles intending to compare control and/or follow-up groups declared a statistically significant difference of data in some domains, with a *p*-value set at *p* < 0.05.

## Domains and instruments

### Oral health-related quality of life (OHRQoL)

OHRQoL was investigated through accepted Oral Health Impact Profile (OHIP) and Child Perceptions Questionnaire (CPQ_11–14_; Jokovic et al. 2002; Slade 1997). The versions of OHIP consisted of either 14 or 49 questions, while CPQ_11–14_ had 16 questions. The questions included different aspects concerning oral function, orofacial pain, orofacial appearance, and psychosocial impact, giving an overall view of OHRQoL. The total mean of OHIP-14 in the studies were compared to control groups, respectively, and shown to differ significantly. Although the mean of the total score of OHRQoL is not standardised, the questions are validated in different languages (Allen and Locker 2002; Hagglin et al. 2007).

In the group receiving crown therapy, OHIP scores and psychosocial and orofacial impact decreased after treatment, indicating higher OHRQoL (Pousette Lundgren et al. 2015).

The OHIP score was significantly higher in the AI group, compared to healthy controls matched by age (Coffield et al. 2005; Pousette Lundgren et al. 2015). No difference was found in rating OHRQoL due to gender among patients with AI (Parekh et al. 2014). Four articles investigated OHRQoL, where all three studies with control groups found patients with AI rating their OHRQoL lower than the controls (Coffield et al. 2005; Hashem et al. 2013; Pousette Lundgren et al. 2015).

The study using OHIP-49 found that patients with AI scored higher in total in the subclasses of physiological discomfort, physical disability, psychological disability, and social disability, compared to the control group (Hashem et al. 2013). In a group receiving crown therapy, the OHIP-14 scores for psychosocial and orofacial impact improved after treatment (Pousette Lundgren et al. 2015). Parekh et al*.* (2014) indicates patients with AI and high CPQ_11–14_ scoring, correlates to lower confidence.

### Dental fear

One study aimed to investigate dental fear. Pousette et al*.* used Children's Fear Survey Schedule-Dental Subscale (CFSS-DS) with a set of 15 items to answer, generating a total score of 15–75 (Klingberg 1994; Pousette Lundgren et al. 2015). The CFSS-DS score in AI patients showed no significant difference, compared to the healthy controls indicating no increased dental fear in patients with AI (Pousette Lundgren et al. 2015).

### Aesthetics

The Coffield et al*.* study used a questionnaire with answers correlating to importance, score ranging *Yes*–*No*. Lindunger et al*.* had a questionnaire answered by VAS ranging 0–10. Chen et al*.* used questions concerning aesthetics answered by the Wong-Baker FACES Pain Rating Scale, a 6-graded FACES-scale ranging from a smiling child’s face to a crying child’s face where the child points to the level of pain, scoring 0–5, where 5 is a crying face (Tomlinson et al. 2010). There is no translation between the instruments used, though the results all indicate a dissatisfaction with esthetics prior to treatment.

The four articles handling aesthetics used two different instruments, questionnaires/questions or interviews. Investigations were performed concerning patients’ own opinions of appearance, i.e., colour, shape, tooth size and the smile (Parekh et al. 2014). The patients’ dental satisfaction after treatment was shown by Lindunger and Smedberg (2005). Chen et al*.* (2013) found patients with amelogenesis interpreting their aesthetics better after dental treatment. Before prosthetic treatment, patients were dissatisfied with their teeth with the discolouration of teeth being a major factor (Lindunger and Smedberg 2005). One article, Lyne et al. (2021) used an AI-specific questionnaire and found appearance and confidence to smile as the most common concerns (76%).

### Psychosocial factors

There were five studies investigating the domain of psychosocial factors, including social interaction and self-esteem. Four of the studies used one instrument, while one of the studies used three instruments for the domain.

Patients with AI showed no difference in reported self-esteem using the Rosenberg Self Esteem Scale (Hashem et al. 2013). Social interaction anxiety, measured by Social Anxiety Disorder (SAD) and Fear of Negative Evaluation (FNE), showed patients with AI scoring higher, indicating more social avoidance and higher social distress (Coffield et al. 2005). Few patients reported teasing from peers, but not necessarily linked to the individuals perception of appearance (Lyne et al. 2021).

There was no difference between genders concerning SAD and FNE in patients with AI, in contrast to the control group (Coffield et al. 2005). Self-esteem and aspects of age in patients with AI were reported to differ from the pattern seen in the general population, where self-esteem is reported higher in young individuals (Coffield et al. 2005). In patients with AI, self-esteem is reported to stay at a constant level through life (Coffield et al. 2005).

The total score of DBS-R did not differ for patients with AI, compared to the control group, and did not change in the study group of AIs before and after dental therapy (Pousette Lundgren et al. 2015). Discussions in focus groups revealed that the patients expressed fewer concerns regarding their appearance, compared to their parents (Sneller et al. 2014).

### Function

The domain function was investigated through questions and answers via Wong-Baker FACES Pain Rating Scale in one study or investigated through a questionnaire and clarified via CPQ_11–14_ by another study. Chen et al*.* showed no difference in response to the patients’ ability to eat before and after dental treatment (Chen et al. 2013). Parekh et al*.* (2014) presented no difference or effect in the oral function for patients with AI.

### Dental hypersensitivity

Six articles investigated dental hypersensitivity using analysing instruments consisting of questions or interviews. The patients either answered the questions through VAS, Wong-Baker FACES pain rating scale, or with *Yes* or *No*, or graded *often, sometimes, never*.

More than 70% of the patients who answered an AI-questionnaire, reported to experience pain or hypersensitivity “often” or “sometimes” Lyne et al. (2021). The dental hypersensitivity was found to be higher in patients with AI compared to the control group (Lundgren and Dahllof 2014; Quandalle et al. 2020). Patients undergoing dental treatment including restorations or crown therapy were found to express a lower degree of dental hypersensitivity (Chen et al. 2013; Lundgren et al. 2018). In interviews, dental hypersensitivity was expressed as a problem (Pousette Lundgren et al. 2016). Among patients with AI, the patients with the hypocalcified type of amelogenesis imperfecta showed a higher frequency of dental hypersensitivity (Quandalle et al. 2020).

### Treatment outcome

The treatment outcome is a domain where studies have analyzed the patient’s report after dental treatment through questionnaires, VAS, interviews, or investigations via OHIP-14, before and after treatment. Restorative intervention shows a positive effect for AI patients, subsequently rating OHRQoL higher, post treatment (Lindunger and Smedberg 2005; Pousette Lundgren et al. 2015). The subclasses in OHIP-14 of orofacial appearance and psychosocial impact were shown to improve after treatment (Pousette Lundgren et al. 2015). A qualitative report showed that dental treatment gives AI patients an improvement in dental situations and a sense of normalization (Pousette Lundgren et al. 2016).

## Discussion

One study limitation is the small number of articles available regarding AI and patient reports, partially due to the diagnosis being rare. Although there are a limited number of PROMs from patients with AI, they all conclude through different aspects how seriously amelogenesis affects the patient’s life. Another limitation is the large variety of methods and approaches seen in the current research making a coherent analysis difficult, since the authors present and measure the impact of OHRQoL in different ways. Statistical comparison of results can be performed in studies including a control group but cannot be achieved in studies of qualitative design nor between the included studies here. All studies confirmed the deep impact on life that patients with AI experience.

This is the first systematic review estimating how the impact of AI is interpreted by patients, which can be considered a strength since it is the first complete summation of the subject.

The systems to categorize AI are well accepted, with subgroups. The subgroups are diverged and have different genotypes and phenotypes (Rao and Witkop 1971; Sundell and Koch 1985). Symptoms from patients are also expressed in various ways and individually. The fact that the prevalence of AI is low does not diminish the effect AI has on daily life.

In dentistry, the domain OHRQoL has previously been correlated to young patients with different dental diagnoses, indicating an impact. Caries is connected to a negative effect on OHRQoL in preschoolers. When the severity of caries is aggravated, a more negative relation to OHRQoL is seen (Nora et al. 2018). Traumatic dental injuries of preschoolers exert a negative impact on OHRQoL, with a need for early dental trauma prevention (Borges et al. 2017). Findings from PROMs, along with odontological science, show that caries and dental traumatic injuries motivate early prevention. The diagnosis of amelogenesis is congenital affecting QoL as soon as the teeth are erupted. No prevention is available for AI, but prevention of caries and other oral pathologies for the patient with AI should be greater. The need for dental treatment at an early age is therefore of extra importance. The requirement for dental treatment is not hard to visualise, though not always implemented due to difficulties to perform in the growing child. The impact of PROMs facilitates a general understanding of the patients with AI. OHRQoL is an important factor to be included when judging and balancing the overall comprehension for the need of early dental treatment.

The OHRQoL reflects the patient experience of oral health and the influence of social interaction, self-esteem, social activities, and performance. The OHRQoL is the patient's interpretation of how the oral region interacts with function of teeth and mouth, symptoms from oral and dental diagnoses, social and emotional mood state of the individual, as well as the connection to the individual’s environment. PROMs, i.e., OHRQoL, are important to increase and widen dental professionals’ understanding for the patients’ well-being and how dental treatment makes a difference (Sischo and Broder 2011).

Overall, patients with AI rate their OHRQoL lower, affecting several aspects of life such as psychological discomfort and physical and social disability. For patients with AI the dental treatment is considered a main factor for improving OHRQoL.

The aesthetic domain of this study presented the patients’ concerns of appearance, e.g., colour, shape and size of teeth (Lindunger and Smedberg 2005; Parekh et al. 2014). The most relevant factor of aesthetic was discolouration of teeth, an element which can be more prominent in the hypocalcified subgroup of AI but should be considered in all patients. Aesthetics is a highly subjective, but none the less important, aspect of treatment.

Patients reported experiences of teasing due to their dental appearance (Coffield et al. 2005; Lyne et al. 2021). Through interviews, many of the participants reported a fear of the teeth falling apart and a feeling of embarrassment over their appearance (Pousette Lundgren et al. 2016). The results, according to patients with AI, revealed a behaviour of avoidance and various ways of dealing with their problems such as making excuses, causing fights, or feeling resignation (Pousette Lundgren et al. 2016). One interesting finding was that AI patients did not report a lower level of self-esteem (Hashem et al. 2013) although other results indicated increased social anxiety and higher social distress (Coffield et al. 2005). These slightly contradictive results enlighten the importance of considering each patient individually, since not all patients have the same experiences and concerns.

Function is a main factor dentists aim for. Chen et al*.* (2013) argued that function may still be limited in patients with AI when no difference in function was seen after restoration treatment, i.e., the patient is still restricted to certain foods due to risk of restorative failure.

Pain and sensation interpreted as dental hypersensitivity is regarded to affect patients’ OHRQoL (Idon et al. 2019). The need for improving tooth sensitivity is one of the major reasons to seek treatment for patients with AI (Parekh et al. 2014). Hypersensitivity is seen to decrease after treatment, indicating the importance of restorative intervention from an early age (Chen et al. 2013; Lundgren et al. 2018).

According to patients, restorative intervention was shown to be important in the retrospective and prospective questionnaires. There are possibilities to improve OHRQoL with modern methods, such as ceramic crowns in early adolescence without a risk for endodontic complications (Lindunger and Smedberg 2005; Lundgren et al. 2018). Bonding techniques have improved and ceramic restorations are a less invasive treatment option to consider for AI patients. Patients with different AI subcategories may have different subjective needs for treatment, highlighting the importance to listen to the individual patient (Lindunger and Smedberg 2005).

Dentistry is part of healthcare, and in this condition where treatment has a great impact on daily life, a subvention system for dental care is welcomed.

### Suggestions for future research

Considering the results of the review, further research concerning PROMs of early interventions in AI patients is desired. AI has a distinct impact on daily life and early dental treatment improves patients’ satisfaction. The professionals’ approach to timing dental treatment needs to be re-evaluated. To make dentists more confident regarding early treatment, studies of *PROMs of early intervention* in AI patients may be helpful.

## Conclusion

Considering any limitations of the present review, it has been shown that the moderate amount of research on PROMs from patients with AI indicates a significant impact on daily life. Patients report a range of concerns such as aesthetics, hypersensitivity, function, and a general impact on well-being and social interaction. This highlights the importance for the need of early dental treatment.
